# When Complex Models Fit the Wrong Mechanistic Complexity in Phylogenomic Analysis

**DOI:** 10.1007/s00239-026-10321-8

**Published:** 2026-05-23

**Authors:** Liang Liu, David A. Liberles

**Affiliations:** 1 Department of Statistics, Institute of Bioinformatics, University of Georgia, Athens, GA USA; 2 Department of Biology, Temple University, Philadelphia, PA USA

**Keywords:** Phylogenomics, Coalescent, Substitution model, Model selection

## Abstract

Substitution model selection is central to phylogenetic inference and is commonly treated as a problem of identifying the substitutional complexity required to describe sequence evolution along a single tree. This framework implicitly assumes a shared genealogy across all sites, an assumption that is routinely violated in phylogenomic data by incomplete lineage sorting and other sources of gene-tree discordance. Recent work by Lozano et al. (2026) demonstrates that unmodeled genealogical heterogeneity can systematically distort substitution model selection, creating spurious support for parameter-rich models even when the underlying substitution process is simple. In this perspective, we examine the consequences of this confounding for phylogenetic estimation, divergence-time inference, and the biological interpretation of substitution model parameters, highlighting the need to more explicitly integrate genealogical heterogeneity into model selection and adequacy assessment in phylogenomics. This perspective reframes substitution model choice not only as a tool for describing molecular evolution, but also as a potential diagnostic for violations of shared-genealogy assumptions that are central to modern systematics.

The choice of an appropriate substitution model is a foundational component of molecular evolutionary inference (Anisimova et al., 2013; Spielman, 2020; Del Amparo and Arenas, 2023). For decades, substitution model selection has been framed as a statistical optimization problem: identifying the stochastic model of nucleotide change that best explains an observed sequence alignment, typically conditional on a fixed tree topology. This practice has become so deeply embedded in phylogenetic workflows that it is often treated as a routine technical step rather than as a potential source of conceptual and inferential uncertainty (Anisimova et al., 2013; Spielman, 2020; Del Amparo and Arenas, 2023).

At the core of conventional model selection frameworks lies an implicit assumption of genealogical homogeneity that all sites within an alignment share a single underlying evolutionary history. Within this framework, heterogeneity among sites is attributed primarily to variation in the substitution process itself, such as differences in evolutionary rates or nucleotide frequencies driven by biochemical constraints, functional roles, or selective pressures (Echave et al., 2016). To accommodate these forms of substitutional heterogeneity while maintaining a single-tree model, phylogenetic methods have progressively adopted increasingly parameter-rich formulations, including gamma-distributed rate variation across sites (Yang, 1993), proportions of invariant sites (Hasegawa et al., 1985), and highly flexible rate matrices (see Woodhams et al., 2015).

The widespread adoption of genome-scale data has, however, called the validity of this single-tree assumption into question. Concatenated multilocus datasets routinely combine sequences drawn from distinct genomic regions that may follow disparate evolutionary histories (Boussau, 2013; Palmer et al., 2019). Processes such as incomplete lineage sorting (Degnan and Rosenberg, 2009; Tan et al., 2023), introgression (Hibbins and Hahn, 2022), hybridization (Kubatko, 2009; Wang et al., 2021), and gene duplication and loss (Rasmussen and Kellis, 2012; Smith and Hahn, 2021; Soewongsono et al., 2025) routinely generate topological discordance among loci, indicating that genealogical heterogeneity is often the biological norm rather than an exceptional complication. Despite this, standard substitution model selection procedures typically disregard underlying conflicts among gene trees. As a result, the selected model is frequently interpreted as a faithful representation of the molecular substitution process, even though it may instead reflect a statistical compromise imposed by unmodeled genealogical discordance.

In this issue, Lozano et al. (2026) explicitly interrogate this long-standing disconnect between model assumptions and empirical reality. By demonstrating that genealogical heterogeneity can systematically bias substitution model selection toward increasingly complex models, their study challenges conventional interpretations of what substitution models capture in concatenated analyses. Their results suggest that, under realistic evolutionary scenarios, model selection criteria may be more sensitive to hidden topological conflict than to true substitutional heterogeneity. This work prompts a reassessment of substitution model selection as an inferential step in phylogenetics and underscores the need to integrate concepts of genealogical heterogeneity more explicitly into systematic practice.

## Model Selection Under a Shared Genealogy

Classical nucleotide substitution models were developed within an explicitly genealogical framework, in which sequence evolution is modeled along a single, fixed tree. Early formulations, including the Jukes–Cantor (Jukes and Cantor, 1969) and Kimura two-parameter models (Kimura, 1980), describe stochastic character change along branches of a shared genealogy, with later extensions accommodating empirical features of molecular evolution such as base-frequency bias (Felsenstein, 1981), transition–transversion and other base conversion asymmetry (Lanave et al., 1984; Woodhams et al., 2015), and rate heterogeneity among sites (Yang, 1993). Importantly, these extensions were motivated by mechanistic and biological considerations pertaining to the substitution process itself, not by differences in underlying tree topology or branch lengths among sites.

Within this framework, likelihood-based and information-theoretic approaches to model selection are conceptually well defined. A more parameter-rich model is preferred when its improvement in fit outweighs the penalty imposed by increased complexity. Under the assumption of a shared genealogy, such improvement is naturally interpreted as evidence that a more realistic description of the substitution process, such as heterogeneous rates or asymmetric substitution patterns, is required to explain the observed data.

Crucially, this interpretation depends on the validity of the shared-tree assumption. When all sites genuinely evolve along the same genealogy, increases in likelihood can be meaningfully attributed to improvements in modeling the substitution process. When this assumption is violated, however, the source of improved model fit becomes ambiguous. Additional parameters may instead compensate for unmodeled genealogical heterogeneity, allowing a single-tree model to better approximate data generated under multiple underlying histories. Under such conditions, model selection no longer provides a clear basis for distinguishing substitutional complexity from violations of shared ancestry.

## Networks vs. Trees

The goal in building a phylogeny is to capture the evolutionary history of the sequences (and in the species context, the organisms they represent). When there isn’t a single topology representing the origin of the samples, one alternative to assuming a fixed genealogy is to model evolution with a phylogenetic network instead of a phylogenetic tree (Bryant and Moulton 2004; Wen et al. 2018). The biological underpinnings of a phylogenetic network is that every nucleotide is represented by a single tree that is embedded in the network such that the network structure is a union of such trees but contains an evolutionary history that is not relevant in all paths to any given nucleotide. That isn’t necessarily a problem. However, methods to build networks using complex model-based approaches and to perform inference on networks, including tests for selection or ancestral state reconstruction are much less developed than for trees. In building methods that are robust to samples with multiple underlying topologies, expanding the methodological toolbox for phylogenetic network construction and analysis is one fruitful direction for the community.

## Genealogical Heterogeneity as a Hidden Axis of Variation

Gene-tree discordance introduces an axis of variation in sequence data that is fundamentally distinct from, yet formally confounded with, traditional substitution model parameters. Even when loci evolve under identical substitution processes, their underlying genealogies may differ in both topology and branch lengths (see for example, Degnan and Rosenberg 2009). When such loci are concatenated and analyzed under a single-tree framework, the resulting alignment represents a mixture of evolutionary histories that no single genealogy can faithfully capture.

Lozano et al. (2026) explicitly examine the consequences of this mismatch between data-generating processes and model assumptions. Using simulations in which all sites evolve under the simplest possible substitution model without rate heterogeneity, compositional bias, or other forms of substitutional complexity, the authors vary only the number of distinct gene trees contributing to a concatenated alignment. As genealogical heterogeneity increases, standard likelihood-based model selection procedures increasingly favor more parameter-rich substitution models, particularly those incorporating among-site rate variation.

This result is notable not because it is unexpected, but because it renders explicit a phenomenon likely pervasive in empirical analyses yet rarely acknowledged. Variation in branch lengths and topologies among gene trees generates patterns of site-wise divergence that, when projected onto a single inferred tree, closely resemble classic signatures of rate heterogeneity. In response, substitution models accommodate this variation by inflating rate-distribution parameters, despite the absence of genuine differences in substitutional tempo among sites.

From this perspective, commonly invoked parameters such as the gamma shape parameter or the proportion of invariant sites assume a dual statistical role. Beyond modeling authentic biochemical or functional heterogeneity in substitution rates, these parameters can also serve as effective summaries of unmodeled genealogical discordance. This duality complicates biological interpretation of model choice and calls into question the extent to which selected substitution models can be straightforwardly construed as descriptions of the underlying molecular evolutionary process.

## Reinterpreting Substitution Model Complexity

The interpretation of substitution model complexity as direct evidence for biological complexity in the substitution process is deeply entrenched in molecular systematics (Steel 2005). Parameter-rich models are commonly viewed as biologically informative, invoked to support inferences about heterogeneous selective pressures, functional constraints, or other forms of site-specific evolutionary variation. The results discussed here urge caution against such straightforward interpretations, particularly in the context of concatenated phylogenomic analyses.

If genealogical heterogeneity alone can drive the preferential selection of complex substitution models, even when the underlying substitution process is homogeneous, then model complexity cannot be uncritically equated with substitutional realism. Rather than indicating a more accurate description of molecular evolution, increased parameterization may instead reflect a mismatch between the assumed genealogical framework and the true data-generating process. Under such circumstances, substitution models absorb variation that originates not from biochemical processes, but from unmodeled discordance among gene trees.

This observation aligns with broader discussions in phylogenetics concerning the distinction between model fit and model adequacy. A model may outperform alternatives according to standard selection criteria while remaining conceptually inadequate if it accommodates violations of key assumptions in unintended ways (Steel 2005; Liberles et al. 2013). Viewed through this lens, increasingly complex substitution models may function as statistical surrogates for missing components of the evolutionary model, most notably, an explicit treatment of genealogical heterogeneity. Recognizing this distinction is essential for interpreting both model choice and downstream phylogenetic inferences in genome-scale analyses.

## Model Adequacy and Model Selection

Model selection is performed using various information criteria that compare the fit of the data by the model with the number of parameters used to do so (see for example, Susko and Roger 2020 for a discussion). Model selection presents a comparison between alternative models that are proposed, as a formalization of scientific hypothesis testing (Liberles et al. 2013). This comes from the perspective that modeling is not meant to just fit data but is an activity that enables discovery of scientific processes. But what happens when all of the models generated are mis-specified? Model selection at this stage still presents the best scientific explanation for the data among hypotheses that have been generated, but one still should know that the model doesn’t explain data well and that some new thinking about process and the model is needed.

How is model adequacy evaluated? The most standard approach in phylogenetics is to perform posterior predictive simulation (Duchene et al. 2019; see McCarthy et al. 2023). However, what happens if a complex model fits the data well but is still mis-specified in that parameters are not fitting what they were mechanistically designed to fit? One may not know that this is the case except when one needs overly complex mixture models to fit data that might provide a red flag. The solution to diagnosing this as a problem is using sets of simulations that make different sets of assumptions and examining the fit of the model to understand when parameters are mopping up complexity that the model isn’t designed to fit. There isn’t a standardized answer to doing this in the absence of specific hypotheses about the biological generation of the data. In this case, the hypothesis that does present itself is the absence of a single underlying tree.

One diagnostic that has been presented in the literature is to ask whether the observed nucleotide site-pattern frequencies are compatible with a homogeneous gene tree model, in which all sites are assumed to evolve along the same underlying genealogy under a specified substitution process (Liu and Yu, 2010). This can be evaluated using classical goodness-of-fit tests, such as the Pearson chi-square test or likelihood ratio tests, which compare observed site-pattern counts to their expectations under the fitted model. In this framework, systematic deviations between observed and expected frequencies indicate that the assumed single-tree model fails to capture important structure in the data. Notably, such lack of fit can arise even when the substitution process itself is correctly specified, if heterogeneous gene genealogies generate site-pattern distributions that cannot be represented by any single tree. Consequently, rejection of the homogeneous gene tree model by these tests should be interpreted cautiously: it may reflect unmodeled genealogical heterogeneity rather than inadequacy of the substitution model per se. Used in this way, goodness-of-fit tests provide a useful diagnostic for violations of shared-genealogy assumptions, complementing information-criterion–based model selection and posterior predictive approaches.

## Implications for Phylogenetic Inference

The implications of genealogically driven substitution model complexity extend well beyond the model selection step itself. Substitution models shape virtually all components of phylogenetic inference, influencing estimates of tree topology, branch lengths, divergence times, and ancestral character states. If selected models are, in part, absorbing genealogical conflict rather than capturing features of the substitution process, then downstream inferences may inherit systematic, but difficult-to-detect, biases.

Inflation of among-site rate heterogeneity parameters provides a clear illustration. Apparent increases in rate variation can distort branch-length estimates, thereby affecting interpretations of relative evolutionary tempo among lineages. Divergence time estimation may be particularly sensitive to such effects, as many molecular clock models interact directly with assumptions about rate heterogeneity across sites (Soubrier et al. 2012; Mello and Schrago 2024). Likewise, tests for molecular adaptation or selection that rely on fitted substitution models may be confounded if inferred complexity reflects genealogical discordance rather than true heterogeneity in selective regimes (Diekmann and Pereira-Leal 2016).

The results of Lozano et al. (2026) also highlight meaningful differences among commonly used information criteria. More conservative criteria penalize over-parameterization more strongly and may therefore be comparatively resistant to model inflation under moderate discordance. Nevertheless, no criterion is immune when genealogical heterogeneity is extreme. This underscores that model selection is not a purely mechanical procedure but reflects trade-offs between goodness of fit and parsimony that interact in nontrivial ways with data structure and model misspecification.

## Concatenation vs. Coalescent

These findings contribute to a growing body of work reexamining the widespread use of concatenation relative to coalescent-based approaches in phylogenomics (Liu et al. 2015). Although concatenation can yield statistically consistent estimates of species relationships under certain conditions, coalescent theory makes clear that heterogeneous gene genealogies are an expected outcome of population-level processes such as incomplete lineage sorting (Liu et al. 2009). The interaction between concatenation and substitution models is therefore more complex than is often acknowledged. The present results suggest that even when concatenation produces a reasonable estimate of species-level topology, it may nonetheless distort inferences about the substitution process itself.

This distortion does not imply that concatenation is inherently invalid, nor that coalescent approaches are universally superior. Rather, it highlights the importance of matching modeling assumptions to the data-generating process. When loci with heterogeneous genealogies, explicitly modeled under the multispecies coalescent, are instead combined into a single alignment and analyzed under a shared-tree assumption, the resulting data violate the generative assumptions of standard substitution models. Under such conditions, improved model fit achieved through increased parameterization may reflect compensation for unmodeled genealogical heterogeneity rather than enhanced realism of the substitution process. Consequently, biologically meaningful interpretation of substitution model parameters requires caution in concatenated analyses lacking an explicit coalescent framework.

## Toward Integrative Modeling Approaches

A central question raised by these findings is how best to accommodate genealogical heterogeneity in phylogenetic inference. One clear solution is greater reliance on models that explicitly account for variation in gene genealogy, such as multispecies coalescent frameworks (Rannala and Yang 2003). By modeling genealogical and substitutional processes separately, these approaches avoid conflating historical discordance with molecular evolutionary dynamics. However, their computational demands and limited integration with flexible substitution model selection currently restrict their routine application to genome-scale datasets.

An alternative direction lies in the development of hybrid or mixture models that incorporate aspects of genealogical heterogeneity while remaining computationally tractable (Liu et al. 2010a, b). Such approaches may capture some of the benefits of coalescent-based modeling without abandoning the concatenation framework entirely. Emerging machine-learning–based tools for model selection and adequacy assessment may also play a role by detecting when increased model complexity reflects deeper violations of shared-genealogy assumptions rather than true substitutional heterogeneity (Liu and Yu, 2010).

From a phylogenetic perspective, for methods not based upon the coalescent, formal gene tree-species tree reconciliation can be undertaken with a model that assumes a particular process is involved in the reconciliation. For example, Rasmussen and Kellis (2012) and Soewongsono et al. (2025) present models that formally characterize the duplication and loss process in reconciling individual gene trees with a species tree, or in the generation of a species tree.

Equally important is a shift in how substitution model selection is interpreted. Rather than viewing model choice solely as a quest for biological realism, it may be more productive to treat it as a diagnostic step. The repeated selection of highly parameterized models, particularly in large concatenated datasets, may serve as an indicator of underlying genealogical conflict, prompting closer examination of data structure and modeling assumptions.

## Broader Implications for Molecular Evolution

Beyond its immediate methodological relevance, this work speaks to a broader challenge in molecular evolution: disentangling multiple, overlapping sources of heterogeneity in genomic data. As phylogenetic datasets increase in scale, they increasingly reflect not only variation in substitution processes, but also complex population-level histories shaped by coalescence, introgression, and lineage sorting. Models developed for simpler evolutionary scenarios often respond by absorbing these complexities into substitution parameters, thereby obscuring the distinction between evolutionary process and statistical pattern.

Recognizing when apparent substitutional complexity is instead a signature of deeper genealogical processes is essential for robust evolutionary interpretation. Lozano et al. (2026) provide a clear and empirically grounded demonstration of this problem, along with a valuable conceptual framework for addressing it.

## Concluding Remarks

By demonstrating that genealogical heterogeneity can systematically bias substitution model selection toward increased complexity, Lozano and colleagues challenge conventional interpretations of model choice in phylogenetics. Their results underscore the importance of aligning modeling assumptions with the structure of genomic data and caution against equating parameter richness with biological realism.

As phylogenomics continues to expand in both scope and complexity, these insights are particularly timely. They serve as a reminder that improved statistical fit does not necessarily imply improved biological understanding, and that substitution models must be evaluated not only by their likelihoods, but also by the assumptions they encode. In this sense, substitution model selection should be viewed not merely as a technical prerequisite, but as a window into the deeper evolutionary structure of molecular data.

## Data Availability

No datasets were generated or analysed during the current study.
